# An exploration of knowledge, attitudes and behaviours of young multiethnic Muslim-majority society in Malaysia in relation to reproductive and premarital sexual practices

**DOI:** 10.1186/1471-2458-12-865

**Published:** 2012-10-11

**Authors:** Li Ping Wong

**Affiliations:** 1 Centre of Population Health (CePH), Department of Social and Preventive Medicine, University of Malaya, Kuala Lumpur, Malaysia; 2 Julius Centre University of Malaya (JCUM), Department of Social and Preventive Medicine, Faculty of Medicine, University of Malaya, Kuala Lumpur, Malaysia

**Keywords:** Knowledge, Attitudes, Young women

## Abstract

**Background:**

The increasing trend of premarital sexual experience and unintended pregnancies in Malaysia warrants sustained and serious attention. The sensitivities of sex-related issues in a Muslim-majority country create various types of barriers to sexual and reproductive health information, support and practices. This study aims to gain understanding of knowledge, attitudes and behaviours of young women in Malaysia concerning reproductive, contraception and premarital sexual practices.

**Methods:**

A cross-sectional study was performed, using an anonymous self-administered questionnaire carried out among 1695 female university students in a public university in Malaysia.

**Results:**

Respondents had low scores for knowledge of reproduction and pregnancy (median=4, of maximum score 10), contraceptive uses (median=6, of maximum score 16) and contraceptive availability (median=3, of maximum score 13). The majority of women surveyed do not have liberal values in relation to premarital sexual behaviour (median=37, of maximum 40); higher scores on this scale corresponded to opposing premarital sex. The multivariate analyses showed that ethnic group was the strongest correlate of knowledge and attitude scores; being of Malay Muslim ethnicity was associated significantly with lower knowledge scores and premarital sex permissiveness. Other significant correlates were year of study, maternal occupational groups, level of religious faith, dating status and urban–rural localities. Level of premarital sex permissiveness was inversely correlated with reproduction and pregnancy knowledge score, and contraceptive knowledge scores.

**Conclusion:**

Reproductive health knowledge and attitudes were intricately linked to religious values and cultural norms differences surrounding sexual issues.

## Background

Sexual reproductive health (SRH) is an increasingly important component of global health [1]. Focusing on young women SRH continues to pose tremendous challenge for policy makers and health care providers. Many young women often lack basic information, knowledge and access to reproductive health services [2]. Parents or guardians are the first and primary sexual health educators of children. Often, because of cultural hurdles, many do not feel comfortable in discussing sex-related issues with their parents [3,4]. Likewise, health care providers and teachers are also reluctant to provide sex-related information to young unmarried people. The reasons for the reluctance were discomfort discussing the issues or erroneous beliefs that providing the information would encourage sexual activity [2,3,5,6].

The reasons why young people engage in sexual activities are complex and diverse and have been attributed to various social contexts and familial factors [7]. Unsafe sex practices among young women and their partners may also stem from lack of knowledge and access to health services for SRH [2,8,9]. Substantial morbidity and social problems among youth are the result of unsafe sex practices resulting in unwanted pregnancies, unsafe abortions and sexually transmitted infections (STIs) including HIV/AIDS [10,11].

Under the influence of mass media, socioeconomic development and modernisation, sexual attitudes and norms have been changing among adolescents and young people in developing [12,13] and Muslim countries [14]. A cross-sectional study in 2001 showed that 5.4% of a total number of 4500 students aged between 12 and 19 years old reported having had sexual intercourse [15]. A national household survey carried out in 1995 by the National Population and Family Development Board (NPFDB) of Malaysia found that about 1% (13 of 1379) of adolescents admitted that they had engaged in sexual intercourse and what was most worrying was that the majority were unprotected sexual encounters [16]. Another nationwide survey conducted in 2005 found that around 1.3% (25 of 1901) of unmarried young people between 15 and 24 years of age were sexually active [17]. Emerging evidence indicates that increasing premarital sexual intercourse resulted in unintended pregnancy among unmarried adolescents and young women in Malaysia. This is evident in the increased number of abandoned baby cases over the years. According to police reports, there were 65 cases of neglected babies in 2010. Between 2005 and 2010, 472 babies were abandoned across the country, of which 258 were dead and 214 were still alive. Premarital sexual relationships are forbidden in Islam and the person who commits the offence of *zina* (sexual intercourse without being validly married to each other) may be punished. Given the familial and social stigma attached to premarital sex and its consequence, and fear of punishment for the offense of *zina*, many resort to abandoning or even killing their newborns.

Although sexual knowledge, attitudes and behaviors among young women have been extensively researched and the literature concerning these issues is abundant, studies related to our unique multicultural and multi-ethnic society, which encompass the Malay, Chinese and Indian ethnic groups, have been few. Understanding of premarital sexual attitudes and behaviours in Eastern, and particularly in Islamic societies, is less detailed. Further, due to the complexity and multifaceted nature of SRH issues, attitudes and perceptions that vary across a broad array of individuals, communities and regions, previous findings may not be applicable to a multi-cultural Asian population and thus studies are warranted.

Malaysia is a multiracial country where Islam is the predominant religion. It is, in many respects, a socially conservative country with regard to reproduction and sexuality. Today, in the era of modernisation, although moderate Muslims form a majority, many still uphold conservative traditional ideas, which consider sex-related issues to be taboo. The challenges of addressing young people’s SRH include various aspects of religious, cultural, social and community attitudes [18]. In Muslim Malaysia, which has a majority of Malay Muslims and sizable Indian and Chinese minorities, family values are defined by an interplay of different religious and cultural beliefs that influence each aspect of an individual’s life. This may in turn frame their behaviours and attitudes towards sexual and reproductive issues. Because of the sensitivity and controversial issues towards the subject, in the past, there was no formal sex education in schools in Malaysia. Only lessons about the human reproductive system were taught in science classes at secondary school level, with little education on sex and safe sex practices. Introducing sex education in public schools in Malaysia caused considerable debate among some conservative groups in the past years. A Social and Reproductive Health Education module, which has a curriculum including biological, socio-cultural, psychological and spiritual aspects of healthy behaviour, was introduced into schools in Malaysia in 2011. Among other subjects, the curriculum covers reproductive systems, conflict management, puberty, the risks of pre-marital sex, sexually transmitted diseases and sexual identity.

### The present study

Given the scarcity of published data on sexual attitudes and behaviour among Malaysian youth in general, the aims of this present study are; 1) to collect preliminary data on the knowledge and attitudes of young women in order to identify information gaps; 2) to assess young women's sexual attitudes and self-reported actual behaviours, and to determine their associations with social/environmental or knowledge factors. The findings, it is hoped, will provide evidence to help develop programmes and policies for improvement of the sexual and reproductive health of youth.

## Methods

### Design and setting

A cross-sectional survey using anonymous self-administered questionnaire. Due to the sensitivity of research queries, the respondents were recruited using purposive sampling. All of the respondents in the study were women from a public university in Klang Valley, Malaysia, who were not taught the Social and Reproductive Health Education module during their secondary school days. The sample was recruited from women attending common areas of the campus during academic semesters between June and October 2011.

### Data collection and measures

The questions in this study represent and build upon existing literature by exploring respondents' knowledge and attitudes about sexual and reproductive health, dating, contraceptive practices and the subjective norms of engaging in risky sexual behaviour (Appendix 1). Questions were arranged in topic areas with less sensitive questions placed first and more sensitive questions placed last. The first part measured personal and family background variables. The second part ascertained the respondents’ knowledge about human reproduction and pregnancy (10 items), awareness of types of contraceptive use (16 methods of contraceptives) and lastly the knowledge of availability or accessibility of contraception (13 items). For the knowledge section, a correct response was given a score of one, and an incorrect or 'don't know' was scored zero; possible total scores ranged from 0 to 10, 0 to 16, 0 to 16, 0 to 13, respectively for reproduction and pregnancy, contraceptive types, contraceptive uses and contraceptive availability knowledge.

The third part consisted of three sub-sections measuring attitudes. All the response options in this section involved the categories 'strongly agree/agree/disagree/strongly disagree', on the scale of 1 to 4. The first subsection investigated respondents’ attitudes towards premarital sex (10-items). The negative statements (supporting premarital sex) were reverse scored, so that higher scores on this scale corresponded to opposing premarital sex. The total possible scores ranged from 10 to 40. The second subsection assessed respondents’ attitudes towards contraceptive use (3 items) and third subsection assessed respondents’ attitudes towards induced abortion (3 items).

The fourth part assessed sources of information and information seeking about sexually-related matters, safe sex practices, contraceptives and pregnancy. The fifth and last section questioned respondents about their first sexual intercourse, enquiring about age of first experience of premarital sex, reasons respondents engaged in first sexual intercourse, the use of contraceptives during the first and later instances of sexual intercourse. The questionnaires were in two languages: Bahasa Malaysia (the national language of Malaysia) and English. The survey questionnaire was tested for face and content validation, and pilot tested.

### Data analysis

The normality of the distribution was assessed by using a Kolmogorov-Smirnov goodness-of-fit test for all the scores. The test indicated that all the scores did not fit a normal distribution. Therefore, non parametric tests were used for the statistical analyses. The Kruskal-Wallis test, a nonparametric test equivalent to the one-way ANOVA, was used for comparisons between the medians of the scores. Chi-square test was used to determine the significance of differences in percentages. Cronbach's alpha was used to assess the internal consistency of knowledge and attitudes scores, with values of at least 0.6 indicating acceptable internal consistency.

Spearman rank correlation coefficients were used to examinethe relationship between knowledge and attitudes scores. All effects were reported as correlation coefficients (*r*). A correlation coefficient (*r*) of 0.10 was defined as of small effect, 0.30 medium effect and 0.50 large effect [19]. For analysis by binary multivariate logistic regression, all the scores were divided into binary variable, dichotomised on the median of each score. Variables were entered into the multivariate model if the univariate P value was < 0.05. The data was analysed using SPSS for Windows (version 17.0, SPSS).

### Ethical considerations

The study was approved by the Medical Ethics Committee of the University of Malaya Medical Centre, Kuala Lumpur, Malaysia. Due care was taken to ensure that all those who agreed to participate in the study did so voluntarily. Written informed consent was obtained from the respondents prior to data collection.

## Results

The background characteristics of the 1695 students who surveyed (response rate 88.1%) is shown in Table 1. The age range was between 17 and 26 years. The internal consistency reliability was good as the Cronbach's alpha of all knowledge and attitude domains were above 0.60, ranging from 0.69 to 0.77.

**Table 1:** Socio-demographic characteristics, and mean total scores of knowledge and attitude (N=1695)

			**Pregnancy knowledge score**		**Contraceptive knowledge score**						**Premarital sex attitude score**	
***Personal background***	**Total N=77**				**Aware**		**Uses**		**Availability**			
			**Range 0-10**		**Range 0-16**		**Range 0-16**		**Range 0-13**		**Range 10-40**	
	**n**	**%**	**Mean/Median**	P^*^	**Mean/Median**	P^*^	**Mean/Median**	P^*^	**Mean/Median**	P^*^	**Mean/Median**	***P***
			**(25**^**th**^**-75**^**th**^**percentile)**		**(25**^**th**^**-75**^**th**^**percentile)**		**(25**^**th**^**-75**^**th**^**percentile)**		**(25**^**th**^**-75**^**th**^**percentile)**		**(25**^**th**^**-75**^**th**^**percentile)**	
**Overall**	1695	100.0	4.3/4.0(3.0-6.0)		8.9/9.0(6.0-12.0)		6.5/6.0(4.0-9.0)		3.7/3.0(1.0-6.0)		35.4/37.0(32.0-39.0)	
***Year of study***												
Pre-university	495	29.2	4.6/5.0(3.0-6.0)		10.4/10.0(8.0-13.0)		7.6/8.0(5.0-11.0)		4.3/4.0(1.0-7.0)		36.1/38.0(34.0-39.0)	
Undergraduate year 1	432	25.5	3.8/4.0(2.0-5.0)	<.001	8.2/8.0(5.0-10.0)	<.001	5.6/5.0(3.0-8.0)	<.001	3.2/2.0(0.0-5.0)	<.001	34.9/37.0(32.0-38.0)	<.001
Undergraduate year 2	332	19.6	4.0/4.0(3.0-6.0)		8.0/8.0(5.0-10.0)		5.6/5.0(3.0-8.0)		3.4/3.0(1.0-5.0)		35.5/37.0(34.0-39.0)	
Undergraduate year 3	436	25.7	4.4/4.0(3.0-6.0)		8.8/8.0(6.0-12.0)		7.0-6.0(4.0-10.0)		4.0/3.0(1.0-6.0)		34.2/36.0(30.0-38.0)	
***Ethnicity***												
Malay	1399	82.5	4.1/4.0(3.0-6.0)		8.8/9.0(6.0-11.0)		6.4/6.0(3.0-9.0)		3.6/3.0(1.0-8.0)		36.0/38.0(34.0-39.0)	
Chinese	213	12.6	5.3/5.0(4.0-7.0)	<.001	10.3/10.0(8.0-13.0)	<.001	8.1/8.0(5.0-11.0)	<.001	4.9/4.0(2.0-8.0)	<.001	31.0/31.0(27.0-38.0)	<.001
Indian	30	1.8	3.5-3.5(2.0-5.0)		8.7/9.0(5.0-12.0)		5.7/5.0(1.0-9.0)		3.1/1.0(0.0-5.0)		34.4/34.0(32.0-37.0)	
Natives of Sabah or Sarawak	53	3.1	3.9-4.0(3.0-4.0)		8.8/9.0(5.0-11.0)		6.6/6.0(4.0-10.0)		3.0/2.0(0.0-5.0)		33.8/34.0(31.0-37.0)	
***Religious faith***												
Very religious	281	16.6	3.8/4.0(2.0-5.0)		8.5/8.0(6.0-11.0)		5.7/5.0(2.0-8.0)		3.2/2.0(0.0-5.0)		36.1/37.0(34.0-39.0)	
Somewhat religious	1349	79.6	4.3/4.0(3.0-6.0)	<.001	9.0/9.0(6.0-12.0)	<.05	6.8/7.0(4.0-10.0)	<.001	3.9/3.0(10–6.0)	ns	35.5/37.0(32.0-39.0)	<.001
Not at all religious	65	3.8	4.8/5.0(3.0-6.5)		9.7/9.0(6.0-14.0)		6.8/6.0(3.0-10.0)		3.3/2.0(1.0-5.0)		31.3/32.0(27.0-37.0)	
***Average household monthly income***^***a***^												
1000 and below	371	21.9	3.7/4.0(2.0-5.0)		8.3/8.0(6.0-11.0)		5.3/5.0(2.0-8.0)		2.9/2.0(0.0-5.0)		36.0/37.0(34.0-39.0)	
1001-3000	729	43.0	4.4/4.0(3.0-6.0)	<.001	9.0/9.0(6.0-12.0)	<.001	6.6/7.0(4.0-9.0)	<.001	3.7/3.0(1.0-6.0)	<.01	35.2/37.0(32.0-39.0)	ns
3001-5000	375	22.1	4.4/4.0(3.0-6.0)		9.3/9.0(6.0-12.0)		7.3/7.0(4.0-11.0)		4.1/3.0(1.0-6.0)		35.0/37.0(32.0-39.0)	
Above 5000	220	13.0	4.3/4.0(3.0-6.0)		9.4/9.0(7.0-12.0)		7.3/7.5(4.0-10.0)		4.1/3.5(1.0-6.0)		35.1/37.0(32.0-38.0)	
***Paternal occupation***^b,c^												
Professional or managerial	720	57.3	4.4/4.0(3.0-6.0)	<.01	9.5/9.0(7.0-12.0)	<.001	7.1/7.0(4.0-10.0)	<.001	4.0/3.0(1.0-6.0)	<.01	35.3/37.0(32.0-39.0)	ns
Skilled workers	537	42.7	4.1/4.0(3.0-6.0)		8.7/9.0(6.0-11.0)		6.1/6.0(3.0-9.0)		3.4/3.0(1.0-5.0)		35.2/37.0(32.0-39.0)	
***Maternal occupation***^b,c^												
Professional or managerial	465	29.2	4.4/4.0(3.0-6.0)		9.5/9.0(7.0-12.0)		7.5/7.0(4.0-11.0)		4.5/4.0(1.0-7.0)		35.5/37.0(32.0-39.0)	
Skilled workers	157	9.9	4.5/5.0(3.0-6.0)	<.01	8.8/9.0(7.0-11.0)	<.01	6.2/6.0(4.0-9.0)	<.001	2.9/2.0(1.0-4.8)	<.001	34.5/36.0(31.0-38.0)	ns
Housewife	970	60.9	4.1/4.0(3.0-6.0)		8.8/9.0(7.0-11.0)		6.3/6.0(3.0-9.0)		3.5/3.0(0.0-5.0)		35.2/37.0(32.0-39.0)	
***Family structure***												
Both parents family	1512	89.2	4.3/4.0(3.0-6.0)		9.0/9.0(6.0-12.0)		6.6/6.0(4.0-9.0)		3.7/3.0(1.0-6.0)		35.4/37.0(32.0-39.0)	
Single parent family	168	9.9	4.3/4.0(3.0-6.0)	ns	8.6/8.0(6.0-11.0)	ns	6.3/6.0(3.0-9.0)	ns	3.5/3.0(1.0-5.0)	ns	35.6/37.0(33.0-39.0)	ns
Guardian/adoptive/parents deceased	15	0.9	3.9/3.0(2.0-6.0)		8.4/8.0(6.0-12.0)		6.0/6.0(2.0-8.0)		3.3/1.0(0.0-6.0)		37.6/38.0(38.0-40.0)	
***Parent’s discipline***												
Permissive	32	1.9	4.5/4.5(3.0-6.5)		10.0/9.5(7.0-14.0)		7.8/8.5(3.0-13.0)		4.6/3.0(1.0-7.8)		32.1/34.0(25.0-38.0)	
Moderate	1124	66.3	4.4/4.0(3.0-6.0)	<.001	9.1/9.0(6.0-12.0)	<.05	6.7/7.0(4.0-10.0)	<.05	3.7/3.0(1.0-6.0)	ns	35.3/37.0(32.0-39.0)	<.001
Strict	539	31.8	3.9/4.0(2.0-5.0)		8.6/9.0(6.0-11.0)		6.1/6.0(3.0-9.0)		3.5/3.0(0.0-5.0)		36.0/37.0(34.0-39.0)	
***Hometown locality***												
Urban	791	46.7	4.5/4.0(3.0-6.0)		9.3/9.0(6.0-12.0)		7.1/7.0(4.0-10.0)		4.2/4.0(1.0-6.0)		35.2/37.0(32.0-39.0)	
Suburban	343	20.2	4.4/4.0(3.0-6.0)	<.001	9.2/9.0(6.0-12.0)	<.001	6.7/7.0(4.0-10.0)	<.001	3.7/3.0(1.0-6.0)	<.001	35.1/37.0(32.0-39.0)	<.01
Rural	561	31.3	3.8/4.0(2.0-5.0)		8.3/8.0(6.0-11.0)		5.7-5.0(3.0-10.0)		2.9/2.0(0.0-5.0)		36.0/38.0(34.0-39.0)	
***Dating status***												
Currently dating	457	27.0	4.5/4.0(3.0-6.0)		8.6/8.0(6.0-11.0)		6.4/6.0(3.0-6.0)		3.4/3.0(1.0-5.0)		35.0/36.0(32.0-38.0)	
Previously dated	461	27.2	4.2/4.0(3.0-6.0)	<.001	9.1/9.0(6.0-12.0)	<.05	6.5/6.0(4.0-6.0)	ns	3.7/3.0(1.0-6.0)	ns	35.5/37.0(33.0-39.0)	<.05
Never dated	777	45.8	4.1/4.0(3.0-6.0)		8.9/9.0(6.0-12.0)		6.7/7.0(4.0-7.0)		3.9/3.0(0.0-6.0)		35.7/37.0(33.0-39.0)	
***Sexual experience***												
Never	1683		4.3/4.0(3.0-6.0)	ns	9.0/9.0(6.0-12.0)	ns	6.6/6.0(3.0-9.0)	ns	3.7/3.0(1.0-6.0)	ns	35.5/37.0(33.0-39.0)	<.001
Ever	12		5.0/5.0(4.0-6.0)		9.7/8.5(7.0-14.0)		7.8/7.0(5.0-9.0)		3.7/3.5(1.0-5.0)		27.2/26.0(22.0-35.0)	

^a^ 1 US Dollar = 3.0 Malaysian Ringgit (MYR).

^b^ Number not total to 1695 due to missing data, refusal, or deceased.

^c^ Small number of *retired*, and *temporary unemployment* responses were not included.

^*^ Kruskal-Wallis *χ*2 test.

### Knowledge

The Cronbach's alpha for the reproduction and pregnancy knowledge scale was 0.70. In the section that assessed the respondents’ knowledge about reproduction and pregnancy, 10.2% (n=173) believed douching can prevent pregnancies. More than half (61.8%, n=1047) believed a woman cannot get pregnant if a partner does not ejaculate. From a possible range of 0–10 of the total 10 items of questions on knowledge about reproduction and pregnancy, the mean total score was 4.3, while the median was 4.0 (Table 1). Reproduction and pregnancy knowledge score increased gradually from undergraduate year 1 to year 3 students. However, the pre-university students scored higher than all the undergraduate year 1 to 3 students. The knowledge varied greatly by ethnicity; scores were higher in the Chinese, followed by Malays, natives of Sabah or Sarawak and Indians. There were also significant differences across socioeconomic groups; with reproduction and pregnancy knowledge declining with lower average household income, low parental and maternal occupational grouping and less urbanised locality grouping. The strength of religious faith and parental discipline strictness were inversely associated with the reproduction and pregnancy knowledge score.

The Cronbach's alpha for the contraceptive awareness scale was 0.73, 0.77 for the contraceptive uses scale, and 0.69 for the contraceptive availability scale. Of the total of 16 methods of contraceptive listed, the majority had heard of at least nine methods. The nine most commonly recognised contraceptive methods in order of most cited were condoms (98.9%), birth control pills (97.8%), withdrawal (81.7%), diaphragm (75.5%), female condom (69.1%), intrauterine device (62.3%), abstinence during fertile times (58.4%), emergency birth control pills (57.7%) and cervical cap (50.8%). As shown in Table 1, the pre-university students scored significantly higher than all the undergraduate year 1 to 3 students in total knowledge of contraceptive methods. Among the undergraduate year 1 to 3 groups, there was a gradual increase of levels of contraceptive methods awareness scores over year 1 to year 3 students. The contraceptive methods awareness scores again vary greatly by ethnicity, being highest among the Chinese. There were also significant differences across socioeconomic groups; awareness scores declined with lower average household income, low parental and maternal occupational grouping, and less urbanised locality grouping. The strength of religious faith and parental discipline were inversely associated with the reproduction and pregnancy knowledge score. Respondents who are currently dating had significantly lower scores of awareness of contraceptive methods than those who previously dated and never dated.

Of the 16 methods of contraceptives, most of the respondents have knowledge of contraceptive use or knew how to practice at least six methods: birth control pills (83.6%), condoms (82.2%), abstinence during fertile times (64.3%), diaphragm (52.6%), female condoms (45.4%) and emergency birth control pills (45.4%). Contraceptive use knowledge score was lowest in the undergraduate year 1 group and gradually increased for each academic year groups. The pre university group presented the highest score. Chinese respondents had significantly higher contraceptive use knowledge score than that of other ethnic groups. Respondents whose parents were employed in professional or managerial occupations, with higher average household monthly income, and lived in urban localities had significantly higher scores. The strength of religious faith and parental discipline were inversely associated with the contraceptive use knowledge score.

The majority knew the availability or accessibility of only three contraceptive methods (condoms 70.4%, birth control pill 69.4% and emergency contraceptive pill 35.2%). Likewise, availability scores were highest among the pre university students, Chinese ethnic group, parents employed in professional or managerial occupations, higher average household monthly income and from urban localities.

The spearman's correlation coefficient in Table 2 shows that all the knowledge scores were positively correlated with each other (*P*<0.01). Specifically, the reproduction and pregnancy knowledge score correlated positively and significantly with the contraceptive awareness score (*r*=0.305) and contraceptive use score (*r*=0.327), with *r* between 0.3 to 0.5, indicates medium effects. The correlation between reproduction and pregnancy knowledge score and contraceptive availability score (*r* = 0.259) indicates small effect. There were significant large-effect size correlations (*r* above 0.5) between 1) contraceptive awareness score and contraceptive use score (*r*=0.688), 2) contraceptive awareness score and contraceptive availability score (*r*=0.540) and 3) contraceptive use score and contraceptive availability score (*r*=0.674).

**Table 2:** **Nonparametric Spearman correlation coefficients (*r*) showing the associations between mean total scores of knowledge and premarital sex attitude (N=77)**

**Mean total score**	**Pregnancy knowledge score**	**Contraceptive knowledge score**			**Premarital sex attitude score**
		**Contraceptive awareness**	**Contraceptive uses**	**Contraceptive availability**	
Pregnancy knowledge		0.305^**^	0.327^**^	0.259^**^	−0.127^**^
Contraceptive awareness			0.688^**^	0.540^**^	−0.072^**^
Contraceptive uses knowledge				0.674^**^	−0.071^**^
Contraceptive availability knowledge					−0.061^*^
Premarital sex attitude					

^**^ correlation is significant at the 0.01 level.

^*^ correlation is significant at the 0.05 level.

### Attitudes

The Cronbach's alpha for the attitudes towards premarital sex scale was 0.74. With higher scores corresponding to opposing premarital sex, the median score of 37 on the possible scoring range 10 to 40 indicated majorities did not have liberal attitudes in relation to premarital sex. As shown in Table 1, the pre-university students scored significantly higher in attitudes towards premarital sexual behaviour compared with the undergraduate year 1 to year 3 students. The score was highest among the Malays (median=38.0) and lowest among the Chinese (median=31.0), with the Indians and natives of Sabah or Sarawak occupying an intermediate position (median=34.0). The premarital sex attitude scores were not significantly different across the socio-economic spectrum (average household income, paternal and maternal occupational grouping). The scores were significantly higher in rural than in urban localities. The attitudes score increased along with increasing degree of religious faith and parental discipline. Currently dating individuals have significantly lower attitudes towards premarital sex scores than individuals who have never dated. Those who had had sex scored significantly lower than who had never had sex. The spearman's correlation coefficient in Table 2 shows that the premarital sex attitudes score was inversely correlated with reproduction and pregnancy knowledge score (*r*=−0.127, *P*<0.001), contraceptive awareness score (*r*=−0.072, *P*<0.01), contraceptive uses score (*r*=−0.071, *P*<0.01) and contraceptive availability score (*r*=−0.061, *P*<0.05). The correlation coefficient of less than 0.1 suggests a weak effect.

Only 2.4% (n=41) and 10.1% (n=171) either agreed or strongly agreed, respectively, that sex with contraceptives indicates less love. Forty-two (2.5%) and 160 persons (9.4%), respectively, either agreed or strongly agreed that women with only one steady sexual partner do not need to use contraceptives. The availability of contraceptives to unmarried youth received slightly higher agreement responses, where 7.2% (n=122) indicated that they agreed and 18.7% (n=317) strongly agreed that contraceptives should be easily available. Only 7.0% either agreed or strongly agreed that pregnant young women should be allowed to get an abortion. More than half (62.4%) either agreed or strongly agreed that abortion should not be allowed in any situation.

### Multivariate logistic regression findings

Table 3 shows the logistic regression coefficients and odds ratios for significant predictors of knowledge and attitudes scores. Undergraduate year 1 students (OR=0.71, 95% CI, 0.68 to 1.39, *P*<0.05; relative to undergraduate year 3), being Chinese (OR=2.33, 95% CI, 1.14 to 4.78, *P*<0.05; relative to being natives of Sabah or Sarawak) and previously dated (OR=1.38, 95% CI, 1.05 to 1.83, *P*<0.05; relative to never dated) were the significant predictors of higher reproduction and pregnancy knowledge score. Pre-university students (OR=2.24, 95% CI, 1.59 to 3.16, P<0.001; relative to undergraduate year 3), undergraduate year 1 students (OR=0.85, 95% CI, 0.40 to 0.86, *P*<0.01; relative to undergraduate year 3) and being Chinese (OR=2.12, 95% CI, 1.03 to 4.36, *P*<0.05; relative to being natives of Sabah or Sarawak) were the significant predictors of higher contraceptive awareness score.

**Table 3:** Multivariate logistic regression analysis models showing significant factors associated to knowledge and attitude scores

	**Pregnancy knowledge score of 5–10*vs*. 0-4**	**Contraceptive awareness score of 10–16*vs.*0-9**	**Contraceptive uses knowledge score of 7–16*vs.*0-6**	**Contraceptive availability knowledge score of 4–13*vs.*0-3**	**Premarital sex attitudes score of 38–40*vs.*10-37**
	**OR (95%CI)**	**OR (95%CI)**	**OR (95%CI)**	**OR (95%CI)**	**OR (95%CI)**
***Year of study***					
Pre-university	1.31(0.93-1.83)	2.24(1.59-3.16) ^***^	1.60(1.14-2.26) ^**^		
Undergraduate year 1	0.71(0.50-0.99)^*^	0.75(0.53-1.06)	0.70(0.50-0.99) ^*^		
Undergraduate year 2	0.97(0.68-1.39)	0.85(0.40-0.86) ^**^	0.55(0.38-0.79) ^*^		
Undergraduate year 3	Reference	Reference	Reference		
***Ethnicity***					
Malay	1.17(0.61-2.26)	0.69(0.36-1.35)	0.77(0.40-1.49)	1.35(0.69-2.67)	3.62(1.68-7.79) ^**^
Chinese	2.33(1.14-4.78) ^*^	2.12(1.03-4.36) ^*^	2.45(1.18-5.10) ^*^	2.87(1.37-6.01) ^**^	0.69(0.29-1.65)
Indian	1.43(0.48-4.30	1.62(0.53-4.91)	1.21(0.40-3.67)	1.02(0.32-3.24)	0.96(0.25-3.68)
Natives of Sabah or Sarawak	Reference	Reference	Reference	Reference	Reference
***Religious faith***					
Very religious			1.50(0.72-3.15)		2.27(0.91-5.67)
Somewhat religious			2.15(1.09-4.25) ^*^		2.82(1.18-6.76) ^*^
Not at all religious			Reference		Reference
***Maternal occupation***^b,c^					
Professional or managerial				1.48(1.12-1.97) ^**^	
Skilled workers				0.71(0.45-1.12)	
Housewife				Reference	
***Hometown locality***					
Urban				1.38(1.02-1.87) ^*^	
Suburban				1.14(0.81-1.61)	
Rural				Reference	
***Dating status***					
Currently dating	1.12(0.84-1.50)		0.69(0.51-0.93)^*^		
Previously dated	1.38(1.05-1.83) ^*^		0.97(0.73-1.29)		
Never dated	Reference		Reference		

^***^ association is significant at the 0.001 level.

^**^ association is significant at the 0.01 level.

^*^ association is significant at the 0.05 level.

With regard to contraceptive use knowledge, as seen in Table 3, year of study was a significant factor, with pre-university students (OR=1.60, 95% CI, 1.14 to 2.26, *P*<0.01) having higher knowledge relative to undergraduate year 3. Chinese students had significantly higher odds (OR=2.45, 95% CI, 1.18 to 5.10, *P*<0.01) relative to the natives of Sabah or Sarawak. The somewhat religious students had higher odds (OR=2.15, 95% CI, 1.09 to 4.25, *P*<0.05) than those who were not at all religious. The currently dating (OR=0.69, 95% CI, 0.51 to 0.93, *P*<0.05) students had significantly lower odds than their respective reference groups.

The multivariate analysis identified being Chinese (relative to natives of Sabah or Sarawak: OR=2.87, 95% CI, 1.37 to 6.01, *P*<0.01), being very religious (OR=2.15, 95% CI, 1.01 to 4.57, *P*<0.05) and somewhat religious (OR=2.47, 95% CI, 1.23 to 4.95, *P*<0.05) relative to not at all religious, individuals whose mothers worked in professional or managerial occupations (relative to housewives: OR=1.48, 95% CI, 1.12 to 1.97, *P*<0.01) and individuals from urban localities (relative to rural: OR=1.38, 95% CI, 1.02 to 1.87, *P*<0.05) as being significantly associated with higher contraceptive availability score.

Being Malay (relative to natives of Sabah or Sarawak: OR=3.62, 95% CI, 1.68 to 7.79, *P*<0.01) and being somewhat religious (relative to not at all religious: OR=2.82, 95% CI, 1.18 to 6.76, *P*<0.05) were the only factors associated with higher premarital sex attitudes score.

### Sexual experiences and contraceptive use

Only 12 respondents reported ever having sexual intercourse, and of these eight were Malays, three Chinese and one native of Sabah or Sarawak. Age at first sexual intercourse was between 17–22 years old. Reasons for having sex included love or physical attraction (one person), pressured by partner (1 person), to build trust in a relationship (five persons), curious and wanting to try sex (nine persons), their own sexual desire (nine persons). Eight of them reported that they did not use any type of birth control during their first sexual encounter. Reasons for not using contraception during a first sexual intercourse were momentary unavailability of contraception (four persons), partner refused to use contraception (four persons) and not wanting to seem as distrusting a partner (two persons). Only four persons reported using contraception consistently over time, with the condom being the most popular method. A total of six persons did not use birth control on most of the occasions when they had sexual intercourse. Factors attributed to non-use of contraception on most occasions of sexual intercourse were lack of access, embarrassment, partner refusal and trusting partners.

### Information seeking and source

Respondents identified magazines (19.2%), friends (18.3%) and teachers (18.3%) as their most common sources of information about sexual intercourse. Only 5.6% of the respondents acquired information about sexual intercourse from their mothers. Respondents were most commonly exposed to information about safe sex practices and contraceptives through magazines (22.4%) and the Internet (20.4%). With regard to information about pregnancy, similarly, the Internet (19.5%) and magazines (19.2%) were the most common source of information.

More than half of the respondents reported that they did not have access to information related to sexual intercourse (67.1%). A considerably large number did not have access to information related to safe sex practices and contraceptives (74.2%) and pregnancy (81.9%) as well. The main reason respondents did not access information about sexual intercourse was embarrassment. The main reason for respondents not seeking information about safe sex practices, contraceptives and pregnancy was that they did not know where to obtain the information. Several respondents noted that the reason for them not seeking information about safe sex practices and contraceptives was because they were not married and did not require the information.

## Discussion

The reproduction and pregnancy knowledge score indicated relatively poor reproduction and pregnancy knowledge among the study respondents. The lack of knowledge about sex and reproduction among youth has been reported in many developing countries [20-22]. Poor knowledge of young people about sexual and reproductive health resulted in youth commencing sexual activity without accurate information about reproductive health, thus putting themselves at risk of engaging in unsafe sexual practices, resulting in STIs or unwanted pregnancies [21,22]. This indicates that women may benefit from SRH education and should not be deprived of it. Fears that sex education encourages or increases sexual conduct have been proved unfounded. Evidence showing that sex education can help delay first intercourse for adolescents [23] should be made known publicly. The notion that higher SRH knowledge does not translate into non-risky sexual behaviour has been agreed upon by many studies [24,25].

The study has shown that while there is good awareness of contraceptive types, knowledge deficits were illuminated in knowing how to use contraception and where to obtain contraceptives. This knowledge deficiency may contribute to the non-use of contraception when they have their first sexual intercourse or inconsistent use of contraception among those in a relationship. The major practical implication of these findings is that reproductive health education should include appropriate teaching about pregnancy prevention, and how to use and to obtain contraception. Much evidence has been presented in favour of contraception education, which helps counter the unfounded myths of premarital contraception teaching and learning among parents and providers. It has been shown that sex education that includes contraception does not increase sexual activity [26], but rather encourages correct and consistent use of contraception for STI protection [21].

The gradient of increasing knowledge scores with increasing family socio-economic status in the univariate analysis indicates that young women with a lower family economic and social status have a higher likelihood of sexual and reproductive health risks, and should be the prime target of future intervention. Findings also pointed out that young women with strict parents were less knowledgeable in both reproductive health and contraception. In our recent in-depth interview study (Wong LP: Qualitative inquiry into premarital sexual behaviours and contraceptive use among multiethnic young people, submitted), parental strictness was found to limit communication about sex in our community. Thus, there is a serious need for communication concerning sexual and reproductive topics between children and parents [27].

The findings of this study are in concordance with the results regarding Muslims in Tehran, Iran, where those who did not consider themselves religious displayed better knowledge about reproductive health than those who labeled themselves as religious [28]. It was reasoned that respondents who reported themselves to be highly religious displayed more traditional cultural sensitivities and religious norms that posed challenges in acquiring SRH knowledge [28]. Given that religious leaders are close to the people in their communities, and are sought to lead on secular issues, it is of priority that reproductive health teaching is integrated in respective national or ethnic religious education programmes. It was also revealed in this study that knowledge was intricately linked to cultural sensitivity surrounding sexual issues. The Malays, the majority of whom are Muslim, had lowest reproductive and contraceptive knowledge scores relative to the Chinese. These ethnic disparities in knowledge scores highlight the need for tailored information on sexual reproductive and contraception for the respective Malay, Chinese, Indian and aborigine of Sabah and Sarawak, respectively. Ethnic-specific reproductive health intervention is important to meet the reproductive and sexual health needs of young women in multiethnic community because knowledge disparities are likely to affect behaviours and, ultimately, reproductive health outcomes among these groups of women.

The fact that pre-university students demonstrated higher reproduction and pregnancy, and contraception knowledge scores than all the other groups may be somewhat due to the pre-university course that covers lessons on Human Reproduction, as part of the Biology course taught though lectures. The undergraduate year 1 to 3, not all of whom had attended the pre-university program, had never had formal sex or human reproduction education, thus, had relatively lower level of knowledge. This is important as it implies that SRH education increased levels of knowledge.

Students who have never dated had significantly lower knowledge on reproduction than those who had dated, which indicates the need to educate young women in general before they begin dating. Earlier school sex education can be beneficial as it was found that earlier lessons on sexual issues was not correlated with earlier onset of sexual intercourse [24]. With regard to knowledge about contraception, particularly worrisome is finding that the group currently in a relationship had significantly lower scores in awareness of contraceptive types. Despite having limited knowledge, they have relatively higher permissive attitudes towards sex.

With regard to attitudes towards premarital sex, our univariate results showed family socio-economic status was not associated with premarital sex attitudes, but rather parental strictness. Although higher parental strictness resulted in a low level of sexual reproduction and contraception knowledge, it has the advantage of contributing to less liberal premarital sex attitudes among our study respondents. Studies in Western countries found that authoritarian control have negative effect on child sexual activities. While parental monitoring can be protective, it was found that youth tend to engage less in sexual activities if parents have a moderate style rather than being overly strict [27]. Therefore, it has been suggested that educating parents about the importance of proper parental monitoring, without intrusion, may ensure low risk of sexual behaviour among youth [27]. In this study, however, strict parenting has positive effect where it yields less liberal attitudes on premarital sexual intercourse. It could be because of traditional Asian-style upbringing that emphasises obedience and respect towards parents. The underlying rationale of this observation is unclear and warrants further investigation to determine if strict Asian parenting should be encouraged in the context of premarital sexual intercourse prevention.

It was found in the study that those who had initiated sexual intercourse hold more liberal attitudes than those who never had intercourse, which indicates that liberal premarital sexual attitudes affect actual sexual behaviour. The impact of the attitude concept and its relationship to behaviour suggests that sustained and traditional socially or culturally constructed conservative premarital sex norms, despite the trend towards more liberalisation in the attitude to premarital sex in modern society is essential.

The most important finding in our study is that we are able to prove that higher reproduction and contraception knowledge do not result in more permissive attitude towards premarital sex. The pre-university students with higher reproduction and pregnancy, and contraception knowledge scores than all the other groups were found to have less permissive attitudes in relation to premarital sexual behaviour. Further, the significant inverse correlation between premarital sexual attitude scores (where higher scores corresponded to opposing premarital sex) and all the other knowledge scores showed that learning about reproduction or pregnancy and contraception does not result in more liberal attitudes on premarital sexual attitudes. This, therefore, may potentially offer essential benefits in prevention of premarital sexual intercourse among young women.

The study respondents themselves disapproved of the availability of contraception for unmarried youth. Such less liberal attitudes to providing contraception to unmarried youth may constitute a barrier to adolescent or young unmarried women contraceptive use. Dealing effectively with contraceptive stigma among unmarried youth requires appropriate sexual health information though numerous channels such as sexual health education in schools, parental guidance, media or reproductive health facilities. Nevertheless, it has been reported that provision of sexual and reproductive health information and services to unmarried young people received unfavourable responses from parents, which resulted in a significant obstacle to the adoption of safe sex practices young unmarried people [29]. Acceptance of provision of SRH services for unmarried young people has not been examined in our local context, but it is anticipated to face obstacles from religious conservatism in our society, as well as parents. A community sex education programme for young people themselves and training parents to be more effective as sex educators of their children seems to be a plausible solution [29,30].

The multivariate analysis showed that being Chinese, older age and with dating experience generally implied an increased knowledge about reproduction and pregnancy. The findings from the multivariate analysis indicated that it is necessary to reinforce contraception education among younger women, the Malay ethnic group, higher level religiosity, no dating experience and rural settings. While being religious was associated with lower level reproduction and contraception knowledge, it appears to provide some protective effect against premarital sexual conduct as exhibited in less liberal attitudes in relation to premarital sexual behaviour. However, it should be noted that there is a possibility of incongruities in expression of attitudes and actual sexual behaviour. Study results from Western countries have been inconsistent, with findings of inverse relationship between religiosity and sexual permissiveness [31], but also others that reported religiosity was not a consistent predictor of premarital sexual attitudes or coital activity [32-34]. Limited evidence emerged from local or Muslim region, thus further studies are needed to support this claim.

Most study respondents identified magazines, the Internet and friends as their most common sources of sexual information. An important source of information, that is, parents, were not identified by many as a source of information on sexual related matters. Positive parent-teen communication has been linked to less risky sexual behaviour among teenagers (Holtzman and Rubinson 1995). These facts emphasise once again the importance of parents in creating a climate for open parent-adolescent communication about sexually related matters at home. Our previous qualitative study (Wong LP: Qualitative inquiry into premarital sexual behaviours and contraceptive use among multiethnic young people, submitted), revealed that many were even less likely to communicate with parents because of the fear that their parents would mistakenly perceive enquiring about sexually-related information as indicating that they had engaged in sexual intercourse. Several factors identified in this study that contributed to lack of access to reproductive and sexual information such as embarrassment, and not knowing where to obtain information warrant urgent action for improving access to and use of information. The survey respondents' belief that safe sex practices and contraceptives information seeking are only necessary for married women should be changed. Women should be encouraged to engage in seeking reproductive information during adolescence. Less than one percent (12 persons) reported that they had already had sexual intercourse could be in part due to under-reporting because of customary sensitivities concerning sexual conduct. The small number of respondents who reported having had sex lead us to being unable to assess correlates of having ever had sexual intercourse and to determine the factors associated with contraceptive use. The key issue indicated by the findings, despite the small number who reported having had sex, was the tendency for non-condom use at first sexual intercourse and on most occasion of sexual intercourse, which exposes one to risk of both unwanted pregnancies and sexually transmitted infections. This finding corresponds to the results of numerous studies; the prevalence of condom use during first sexual intercourse was low among young people [35-37]. It has been suggested that intervention designed to increase condom use among young women to prevent sexually transmitted infections and unintended pregnancies is warranted and, in particular, promotion of condom use at first intercourse is important as it has been shown to predict future condom use [38].

As also noted by many other studies [39,40], the young women in this study faced substantial physical, social and psychological barriers to accessing contraception. Lack of access to contraception indicates that availability or accessibility to contraception is critical in increasing the chances of young women using the method when needed. Therefore, a comprehensive approach towards eliminating all factors that may deprive youth of access to reproductive services is required. Conventional family planning services designed for married women should also try to provide services for the young [40]. The concern that providing contraceptive services may promote premarital sexual intercourse can be overcome by developing specific skills for counselling the young [41].

In many Asian cultures, reproductive health decision-making is based upon male authority and power. The fact that girls want to be perceived as obedient to partners’ refusal to use contraception indicates male dominance over female’s choice of contraception decision-making in this study sample. Studies have shown that women with less power to refuse sex or to insist on condom use relative to their male partners are more likely to have unprotected intercourse [42], and male dominance in decision-making has been reported to hinder the ability of women to practice family planning [43]. Young women were encouraged to have more enlightened and contraceptive-conscious attitudes [44], to increase communication with partners about contraceptives [45,46], to increase effective contraceptive use. It is imperative that contraceptive and reproductive health education is reinforced among males as support from male partners can help to increase contraceptive use and to decrease the likelihood of adolescent pregnancy [45,47]. Asian women should understand that male-dominant decision-making in sexual and reproductive health matters puts women at a disadvantage. Gender stereotypes of submissive females and powerful males can make it impossible for women to refuse unwanted or unprotected sex, and to negotiate condom use. Therefore, it may be beneficial to teach men to reject the ideology of traditional masculinity and advocate the attitude of liberalism towards SRH matter, for the benefit of both males and their partners.

It is particularly worrisome that some reported that they did not use any contraception during a first sexual intercourse because they trust their partners. This may imply that the women in this study relied on “know your partner” or the Implicit Personality Theory in assessing the riskiness of partners [48], which perceived knowing partners’ nonpromiscuous sexual history as reason for unprotected intercourse. It has been suggested that intervention efforts must emphasise that one is not invulnerable to infections, and not practicing safer sex with a partner they trust or whom they perceive might not be risky as in the Implicit Personality Theory may put them at risk of the negative consequences of unprotected sexual intercourse [48].

Considering the embarrassment of procuring condoms, behavioural intervention to cultivate positive attitudes on condom acquisition and to overcome embarrassment over condom purchases is needed. Thus, there is a need to expand access to youth-friendly SRH services to cater for contraceptive needs of young people [49]. Given the reasons for social taboos surrounding SRH issues and social disapproval of premarital sex in Muslim countries like Malaysia, unmarried young people may be embarrassed about acquiring contraception or accessing reproductive health educational information and services. Therefore, the basic components of reproductive services should include specially trained providers, privacy and confidentiality of services provided.

In interpreting these results, there are certain limitations in the study design that might impact upon the conclusions drawn. The major limitation of this self-report behavioural questionnaire is socially-desirable response bias. Given that premarital sex is culturally unacceptable in Malaysia, it is not possible to determine whether the students in this study tended to under-report their sexual experience. Additionally, because the study design was cross-sectional, results can only be considered exploratory and the directionality cannot be assessed. Another significant drawback is the use of purposive sampling, which limits generalisation about the entire population. The strength of this study lies in its large sample of young women obtained from one of the largest public universities in Malaysia; students were from across the whole country and offered a demographically representative sample for the study.

## Conclusion

Young women in societies with conservative norms for sexual behaviour need better information on reproductive health, pregnancy and contraception. Level of knowledge was found to be intricately linked to religious values and cultural sensitivities surrounding sexual issues. Knowledge disparities were also closely linked to ethnic, social, economic and parental factors. Greater knowledge about reproduction, pregnancy and contraception were not associated with more permissive values on premarital sexual behaviours.

The study results have both theoretical and empirical implications for future research. The key findings discussed above draw attention to the importance of providing information to young women before sexual activity commences. There is a need to refute actively common erroneous beliefs that provision of sexual and reproductive health information and services to unmarried young people do not lead to increase in liberal attitudes towards premarital sex. In particular, in a context of both a multiethnic and multi-religious society, ethnic-specific reproductive health intervention is important to meet the reproductive and sexual health needs of young women of different cultural, ethical and religious values.

## Competing interests

The author has declared that no competing interests exist.

## Author’s contribution

WLP planned and organized the data collection, carried out data analysis, drafted and revised the manuscript.

## Pre-publication history

The pre-publication history for this paper can be accessed here:

http://www.biomedcentral.com/1471-2458/12/865/prepub
